# Turn-over and Retention Among Head Start Educators

**DOI:** 10.1007/s10643-024-01685-x

**Published:** 2024-05-16

**Authors:** Delia Vicente, Melanie Venegas, Alma D. Guerrero

**Affiliations:** 1 Development Behavioral Pediatrics Division, Department of Pediatrics, David Geffen School of Medicine at University of California, Los Angeles, CA, USA; 2 Department of Pediatrics, UCLA Mattel Children’s Hospital and Children’s Discovery & Innovation Institute, David Geffen School of Medicine at University of California, Los Angeles, CA, USA

**Keywords:** Early childhood education, Head Start, Workforce, Educator perceptions, Retention, Professional development, Turn-over

## Abstract

Educators shape the quality of early education programs and are essential to children’s learning and healthy development. However, the early childhood education field is often challenged in retaining educational staff. Using a descriptive research design this study explores turnover and retention through the voices of Head Start and Early Head Start education staff. Education staff identified retention factors to be, holding a job with meaning and purpose that made a positive difference for children, families and communities, access to professional development opportunities, and positive connections with colleagues. While, low wages, high volumes of paperwork, complex caseloads, lack of recognition, weak onboarding, teacher preparation practices, and lack of voice in program changes, were identified as turnover factors. Despite low wages educators voiced they are more likely to stay when their growth and relatedness needs are being met. Overall turnover and retention were influenced by an organization’s systems, practices, and working conditions and how well these met the human core needs of its staff.

## Introduction

Educators shape the quality of early education programs and are essential to children’s learning and healthy development. Children who attend high-quality early education settings are more likely to meet school expectations, adapt to school demands, perform better in math and reading, have advanced language skills, have positive self-perceptions and attitudes, and have closer relationships with educators (Cannon et al., 2018; Hale-Jinks et al., 2006; Magnuson et al., 2004). Often high-quality early education settings are defined with structural features, such as teacher child ratios, physical environment characteristics, and teacher qualification (Burchinal, 2018; Pianta et al., 2005; Thomason & La Paro, 2009; Slot et al., 2015). However, quality also includes *process quality* makers typically defined by the child’s experience in early education environments led by teachers such as, the teacher–child interactions, and child’s experience with the instructional aspect of the classroom and materials (Burchinal, 2018; Howes et al., 2008; Thomason & La Paro, 2009). A growing amount of literature suggests that the quality of the teacher–child interactions fosters child development, predicts higher social competence, language, reading, math skills, and lower levels of behavior problems (Burchinal et al., 2010; Pianta et al., 2016). Not surprisingly, retention of educators is a quality marker of early education programs (Bryant et al., 2023; Mims et al., 2008). Yet, the early childhood education field is often challenged in retaining educational staff.

Internationally turnover rates among early childhood educators range from 18% to 47%, in the U.S. it is 33% (Amadon et al., 2023; Hayes, 2004; Thorpe et al., 2021), and federally funded Head Start and Early Head Start programs are not immune to this problem (Bellows et al., 2022; McCormick et al., 2022). One-third of center-based early childhood educators in 2017–2018 were no longer teaching in the same program the following year (Bassok et al., 2021), and for new hires, almost 40% of teachers quit around mid-year without completing the year (Wells, 2015). According to the Center for Study of Child Care Employment at the University of California, Berkeley, the early education industry experienced large job losses during the COVID-19 pandemic and has struggled to recover (McLean et al., 2021). Thus, turnover among education staff in early education settings is a growing concern. Through the voices of Head Start and Early Head Start teaching staff we explored turnover and retention in this descriptive study.

### Turnover and Children

Twelve million children in the United States receive care by teachers in early education settings, such as, childcare centers or preschools (Whitebook et al., 2018), and of these close to a million attend the largest early education program of Head Start and Early Head Start (National Head Start Association, 2023). Therefore, many children are expected to spend a substantial number of hours in educational settings during critical years of health and development (Cui & Natzke, 2021). It is well known that early experiences in the first few years of life are important for brain development and have long lasting influence on child development (Mertz, 2017; Noble, 2021; Tierney & Nelson, 2009, Shonkoff et al., 2000). Many children will be relying on their teacher or early childhood educator for support during this critical time (the term educator will be used interchangeably with teacher or anyone in a teaching position). Studies have identified that quality teacher–child interactions is important and can support positive experiences, learning, and skill acquisition (Pianta & Stuhlman, 2004). Therefore, disruptions to the learning environment such as educator turnover is a substantial risk to child health and developmental outcomes (Bratsch-Hines et al., 2020; Sandstrom & Huerta, 2013).

The negative impact of turnover on children’s health and learning has been well-described, in particular for those from under-resourced communities (Lee & Burkam, 2002). Teacher turnover can negatively impact children by disrupting routines, leaving children confused and longing for a teacher to return, and disrupting the teacher–child attachment, which can lead to emotional stress in children (Cassidy et al., 2011; Hale-Jinks et al., 2006). In addition, teacher turnover has been shown to be negatively linked with child cognition, language, and social-emotional development (Tran & Winsler, 2011). Furthermore, children in Head Start programs that experience high teacher turnover have lower gains in vocabulary and literacy skills (Markowitz, 2024. In contrast, early childhood educator stability and training to support classroom behavior management can promote overall school readiness by improving social and math skills, early literacy, levels of attention and impulsivity, executive functioning, and socio-emotional development (Bratsch-Hines et al., 2020; McCoy et al., 2019; Watts et al., 2018). Turnover also produces a “domino effect” on staff, who are placed into new environments or classrooms without much support which creates additional workload for teachers, floaters, and directors (Cassidy et al., 2011; Whitebook & Sakai, 2003). In addition, staff turnover is expensive, costing an estimated $20,000 to replace each education staff position (Carver-Thomas & Darling-Hammond, 2017). Sorensen and Ladd (2020) have found that high rates of teacher turnover have negative effects on instructional quality, student learning, and the school’s ability to develop strong educational programs. Thus, high turnover rates among early childhood educators have important implications not only for children, parents, education staff, and leadership, but also for the economy since parents need a reliable source of early childhood care in order to work.

### Why Do Educators Leave?

Studies to date indicate that factors leading to educator turnover in early childhood education centers include economic factors, such as low salaries, benefits, and dissatisfaction with wages (Caven et al., 2021; Goelman & Guo, 1998; Phillips et al., 2000, 2016; Schaack et al., 2020, 2022; Whitebook & Bellm, 1999). In particular, low paid teachers with post-secondary degrees are prone to leave to higher paying opportunities (Schaack et al., 2020). Other reasons for turnover are organizational issues such as poor working conditions, ambiguous job descriptions, decreased communication, lack of social support, high workload, and physical and emotional exhaustion (Grant et al., 2019; Rentzou, 2012). Qualitative studies have described similar findings on reasons for turnover. Among the Head Start workforce, work intensification, compensation, workplace and personal stress, and workplace relationships were the top drivers of turnover (Bullough et al., 2012, 2014; Cassidy et al., 2011; Wells, 2017; Wiltshire, 2023). A mismatch between job expectations and adequate professional development resources, work demands, and low wages are also reasons for non-Head Start early childhood educator turnover (Schaack et al., 2020, 2022). Most recently, the COVID-19 pandemic has had a significant impact on teacher turnover due to increased work intensity, and heightened work-related stress (emotional drainage, fatigue, frustration), anxiety, depression, and financial burdens among early childhood educators (Bryant et al., 2023; Souto-Manning & Melvin, 2022).

### Educator Workforce Crisis

The need to improve early education working conditions, including pay, has been known for many decades (Barnett, 2003; Caven et al., 2021; Hale-Jinks et al., 2006; McLean et al., 2019; McLean et al., 2021; Phillips et al., 2016; Totenhagen et al., 2016; Whitebook et al., 2014; Whitebook & Sakai, 2003), but it has been ignored, pushing the early childhood field into a workforce breaking point (National Association for the Education of Young Children, 2022; National Head Start Association, 2022). Unaddressed low compensation continues to drive turnover and is further exacerbated by state minimum wage laws or cost-of-living city ordinances for other workforce sectors. New policies supporting the expansion of universal pre-k models or transitional kindergarten may also contribute to future increases in turnover among highly qualified staff working with young children due to higher salary opportunities offered by local elementary schools (Calderon, 2024). Highly trained teachers in particular those with post-secondary degrees tend to leave programs with low wages (Schaack et al., 2020; Whitebook & Sakai, 2003) to move to higher paying opportunities. Recent assessment of workforce issues conducted by the National Head Start Association (2022) showed turnover was higher than in previous years, with approximately 19% of positions unfilled nationwide.

### Research Aim

Addressing the complexity and implications of turnover also merits research on the converse. As such the current study asks, “Why do Head Start education staff stay in their roles?” To our knowledge, fewer studies have focused on retention drivers and even fewer focused on the live experiences of Head Start educators. Addressing this gap in the literature is important to build upon the positive attributes of the position of education staff. Working to address the core reasons for turnover or low retention rates among programs, while simultaneously leveraging the positive attributes of education staff are both needed to effectively address shortages, closed classrooms, undue workloads for others, and access to high-quality early childhood education programs. Therefore, in the current descriptive study, we explore retention and turnover through the voices of Head Start and Early Head Start education staff using secondary analysis of quantitative and qualitative data and applying a framework of the General Systems and Alderfer’s Existence, Relatedness, and Growth (ERG) theory.

### Theoretical Frameworks

We draw from General Systems and ERG theories to conceptualize turnover and retention (Alderfer, 1969; Kast & Rosenzweig, 1972). General System theory suggests that an organization is composed of various interdependent parts, where any change in one part of the organization will impact another (Kast & Rosenzweig, 1972). Further, open system organizations are influenced by larger environments (Shafritz et al., 2015). Head Start programs follow these theoretical concepts. For example, Head Start programs have multiple interdependent areas of focus including enrollment, health, nutrition, family functioning, disability, mental health, education, tracking, monitoring, and governance among others (Improving Head Start for School Readiness Act 2007). At the same time, Head Start programs are tasked to respond to shifts in the early childhood landscape, due to changes in policy, regulation, community need, or Head Start priorities. These macro-level shifts trigger a domino effect that may impact program administration and contribute to fluctuations in work environments for staff by increasing workloads, training needs, and documentation practices, among other changes without reducing the already existing heavy workload and demands. This end result, therefore, can shape an environment that may discourage retention and encourage turnover of Head Start staff.

Another way to conceptualize retention and turnover of Head Start staff is to consider ERG’s theory which posits that basic human needs and motivation, through the fulfillment of *existential, relatedness and growth* needs, must be met (Alderfer, 1969). In this sense, *existence* needs are physiologic in nature, like having access to food, water, clothing, and safety. *Relatedness* refers to the human need for connectivity and relationships. *Growth* needs are fulfilled through productivity and creativity. Collectively, “these needs provide the basic elements of motivation” (Alderfer, 1969 p.145). For example, a Head Start employee would enter the workplace seeking adequate wages to meet their physiological needs of food, water, clothing, as well as health benefits, and a safe work environment. In terms of relatedness, staff would seek to fulfill the need to connect with others through work relationships while providing an opportunity to share ideas, connect, and feel valued through relationships in the workplace. Last, growth needs would take shape by providing an employee the opportunity to express their creativity, be challenged with tasks, learn new skills, and problem solve to positively influence the work environment and in order to reach full potential of self (Alderfer, 1969). Each person places value on each need differently, and the needs are dynamic and often shifting throughout the lifespan based on life events. When the organization fails to meet the existential, relatedness or growth needs this may play a role in turnover, or inversely if an organization is fulfilling an employee’s needs, this may lead to retention.

## Methods

This study aimed to explore retention and turnover through the voices of Head Start and Early Head Start education staff by following a descriptive study design using secondary analysis of qualitative and quantitative data. For this study, we analyzed data from two distinct set of participants: de-identified transcripts of two focus groups conducted by a Head Start Association and a survey conducted by leadership in a workforce learning collaborative to understand additional views on turnover and retention. This study used secondary data made available to the researchers due to their work previous work in Head Start workforce committee and training collaborative. The UCLA IRB board determined a review was not necessary since the study only included an analysis of deidentified data, known as secondary data analysis. Data sets received did not contain any identifiable information.

### Survey Data Participants

A survey was distributed in October of 2021 to a total of 24 potential (EHS and HS) programs from various states across the United States. Approximately 67% of the programs responded to the survey (16 of 24) which aligns well with national response rates of surveys in social science (65%) and exceeds response rates (44%) of online surveys in published research (Holtom et al., 2022; Wu et al., 2022). The majority of participants were directors (52%), followed by supervisors (16%) and managers (13%).

### Focus Group Participants

Twenty-three Head Start and Early Head Start education staff participated in two focus groups in 2019 facilitated by the same person. They represented 16 Head Start programs in a Western state of the United States. On average, the education staff had 8.7 years of working history with Head Start (median of five years) and 18.9 years of experience in early childhood education (median of 17), see Table 1. Of the total 23 participants in the focus groups, 21 were female; the racial and ethnic composition included, 35% Hispanic/Latinx, 31% White, 26% Black/African American, 4% Asian, and 4% American Indian. The education of the participants ranged from some college to graduate level; 52% had a bachelor’s degree, 17% had a graduate degree, 22% had an associate degree, and 9% had a child development permit and were working towards an associate degree. The study included staff from Head Start programs implemented by non-profits (52%), local education agencies (43%), and Tribal programs (4%).

### Focus Group Data Collection

Focus group data were collected by a Head Start Association from twenty-three Head Start and Early Head Start education staff members in 16 programs in a state in Western United States in 2019. Focus groups met in person, they were recorded, transcribed, and de-identified by the Head Start Association staff and consultants. The de-identified transcripts without names or identifiable information were accessed by the research team and used to complete a qualitative analysis. The study was reviewed by the research team’s Institutional Review Board (IRB) and found to be exempt for review.

### Survey Data Collection

Survey data were collected from a distinct and separate set of participants that included 24 Early Head Start and Head Start (EHS/HS) program directors, supervisors, and managers from various states across the United States who participated in a workforce learning collaborative. An online survey was sent out to the 24 Head Start and Early Head Start directors and administrators participating in this learning collaborative that met monthly from May 2021 through October 2021, during the middle of the COVID-19 pandemic. The questions for the survey were formulated based on shared challenges verbalized by directors and administrative leaders of Head Start or Early Head Start programs during their participation in the monthly collaborative meetings. Participants created self-generated identification codes from answers to a series of personally salient questions to ensure unique and unduplicated responses (i.e., last letter of first name), and informed that by answering and submitting the survey responses consent was provided. The study was reviewed and deemed exempt from obtaining IRB approval.

### Focus Group Measures

Focus group participants discussed their experiences as educators. An outline of open-ended questions was used, and elaboration was sought as topics arose. The focus groups were designed to explore the areas of why participants chose to start working with Head Start as educators, satisfaction, and dissatisfaction with the Head Start workplace environment, drivers of retention and turnover in the Head Start organization, and whether they had the intention to stay or leave their current Head Start/Early Head Start program. The same outline of open-ended questions was used for both focus groups.

### Quantitative Measures

The survey included a total of seven core questions with follow-up questions if core questions were answered in the affirmative. For example, participants were asked, “Do you have staff shortages?” If participants responded “yes” then a subsequent question asked reasons for staff not returning with a list of reasons (i.e., death/illness, higher pay, childcare responsibilities, to avoid/minimize risk of COVID-19 exposure) while also including “other” as a potential choice with free-text answer. Items on the survey also asked respondents to estimate staff shortages (type and/or position) and classroom closures and level of confidence about having full enrollment of children for the upcoming fall. The complete list of items on the survey included the following: (1) What is your current position at your corresponding EHS/HS site? (2) Do you currently have any staff position shortages? If yes, what percentage of the following staff positions have been vacant at your EHS/HS agency in the last month, 6-months, and 12-months? (3) Do you have open and available EHS/HS slots for families at your agency? If yes, what percentage of available slots for infants of children were not filled at your EHS/HS agency last month, 6-months ago, 12 months ago? (4) What percentage of classrooms have not “opened” at your EHS/HS agency in the last month? 6 months ago, 12 months ago? (5) Has a parent communicated a reason as to why his/her child will not be returning to your EHS/HS agency this upcoming fall? If yes, what reason was provided? (6) Has a staff member communicated a reason as to why his/her own child will not be returning to school? If yes, what reason was provided? and (7) Please indicate your level of agreement of disagreement with the following statement: I am confident my EHS/HS agency will be at full infant/child enrollment by January 2022, (1–5 Likert Scale).

### Data Analysis

The de-identified focus group transcripts were shared with the study team for analysis one year after the data were collected. Two members of the study team reviewed the de-identified transcripts and developed a codebook using consensus-seeking iterative discussions. Themes were identified using an iterative, consensus-seeking inductive content analysis approach (Hsieh & Shannon, 2005). The survey data were analyzed using descriptive statistics. Inferential statistics were not used since the intent of this study was to describe turnover and retention through the voices of Head Start staff rather than find associations or predictions.

## Finding and Results

### Focus Groups Findings

Qualitative analysis of focus groups revealed two major themes with accompanying subthemes: (1) retention characteristics, and (2) turnover drivers. Several subthemes were also identified and are described in further detail below.

### Retention Characteristics

Staff educators reported several factors that influenced their decision to stay within Head Start including: (1) positive community impact on children and families, (2) connection with colleagues, and (3) professional development opportunities.

#### Theme 1: Community Impact on Children and Families

Head Start educators who foresaw staying in their position for the next five years cited their perceived positive contribution towards community impact on children and families of the Head Start program as the primary reason they would remain within the organization. Educators expressed passion and commitment about their work, felt they were fulfilling their calling, and a belief that their job was making a difference in the lives of children and families. Educators shared the belief that the Head Start program was attentive to families’ needs in the community and gave children the skills needed to succeed in school. They commended Head Start’s multidisciplinary approach to fulfilling families’ goals in education, healthcare, dental care, mental health, and community resources. An educator stated, “…not only are we impacting the children, we’re impacting the family and the community as well.” They stated their impact had crossed generations, as several Head Start alumni became educators, and parents became Head Start employees. Overall, educators expressed a deep commitment to the Head Start mission and this commitment prompted them to stay working for Head Start/Early Head Start.

#### Theme 2: Connections with Colleagues

The second retention factor identified was a positive connection with colleagues. The staff overwhelmingly valued the positive workplace culture and relationships with their coworkers. Shared physical proximity and frequent interactions facilitated close relationships with on-site staff, including direct supervisors and directors. Any switch or move of an educator to a new teaching team was described as a negative experience, including feelings of sadness and anger for the change. Educators also reported appreciating social events and celebrations with their colleagues, and they felt it was an essential factor in ensuring a warm, welcoming, and inclusive work environment. One participant stated, “…we feel like we are all very much a family inside our building…and we very much want to make sure everyone feels included…”.

#### Theme 3: Professional Development

A third retention factor cited was the availability of professional development opportunities. Educators valued the professional development opportunities available through workshops, job-specific trainings, mentorship, and coaching events. Some educators shared coming into the field seeking career growth opportunities and opportunities to purse educational expense reimbursement. Many educators expressed deep gratitude for the educational experiences and training provided by their organization. When educators were asked about their future career prospects many educators viewed their current position in Head Start as a long-term profession ignited by their passion to make a difference. Educators also shared that their gained experiences being hands-on in the field of early education has allowed them to be well-prepared for their future endeavors. They stated that Head Start provided many opportunities to grow their careers but recognizing the tradeoffs of leaving the classroom and children in order to pursue a new career opportunity, and many were troubled by this notion, “I think that as far as advancement for teachers, unfortunately, to go up and advance it forces you to leave the classroom. If you are passionate about the children and want to stay and be there for them, then it can be a difficult choice.” Educators reported that many times they do not want to leave the classroom but are forced to if they want to earn more money.

### Drivers of Turnover

The education staff identified several turnover drivers including: (1) low wages and benefits, (2) high volumes of paperwork, (3) high and complex caseloads, (4) lack of recognition from the K-12 education system, (5) weak onboarding and training practices, and (6) limited voice and participation in organizational changes.

#### Theme 1: Low Wages and Benefits

All educators in the focus groups reported receiving low wages. The survey data corroborated these sentiments with directors and administrative leadership reporting that EHS/HS staff left their jobs to pursue higher paying jobs. Qualitative data further described receiving wages that were insufficient to absorb the cost of living in their state, and many had multiple jobs to make ends meet. One educator stated, “Our staff are so poor. Sometimes they are struggling as much as the families that they’re serving.” Another educator shared, “The passion is still there…But what other people are not saying is that for us as Head Start teachers, and personally me, I have a second job because I could not live with just that.” When asked about a second job, 75% of educators in the focus group had a second job, and many a third. Many educators reported that the low wages made them consider leaving Head Start, “That’s where it becomes so hard because we’re trying to take care of other people’s families when we can barely take care of our own.” Strategies to counter the low salary of educational staff included seeking supervisory or administrative positions internally within their Head Start program, or often looking for work elsewhere.

Educators reported feeling there was a big pay gap compared to K-12 teachers despite the similar work in teaching young children. They also voiced a need for equality in pay based on educational degrees and experience. Some participants reported returning to school to fulfill the Head Start minimum qualifications for a teacher position, but the increase in education did not necessarily lead to an increase in salary. Participants felt that if a salary increase was not provided when teachers increased their qualifications, they typically left Head Start. A participant stated “…we are losing really good, fantastic teachers because they’re going to the program that pays more money.”

In addition to low salary, several educators reported not being able to afford the medical benefits offered or the ability to add their family members due to cost. A work-around to address the lack of affordable medical benefits for some educators included working part-time instead of full time in order to qualify for state’s Medicaid public insurance program. Lastly, participants also voiced a lack of childcare. Despite earning low wages, educators did not qualify to have their own children enrolled in Head Start or other low-cost child-care programs.

#### Theme 2: High Volumes of Paperwork

Educators cited having excessive and unnecessary paperwork, and an increasing number of child observations and assessments. Some teachers mentioned paperwork took away from the primary goal of teaching children, “….so you have this paperwork that piles up, up, up that eventually you feel like the paperwork becomes more important than the children.” Most educators referenced having to work during their breaks, lunch, and while at home. An educator stated, “I think too much paperwork is expected out of whatever little office time we get.” Participants reported yearly increases in the amount of paperwork. Educators reported that by complying with the ever-increasing paperwork, enabled by a hard-work ethic and time spent outside of the office, they may have inadvertently sent the wrong message to their management team with an incorrect assumption that educators could handle additional paperwork and forms to complete. Despite the large amount of paperwork, most completed it, “…teachers keep fulfilling the paperwork, we keep doing it, so I think that they’re [under] the perception that, ‘Oh, we can add one more [form]. They can handle it.’” Educators expressed that upper management and supervisors were unaware of how often staff used their personal time to complete all required paperwork, a pattern that prompts many to leave the job. A participant stated,” So I think Head Start has a high turnover rate for employees because one, we go somewhere [to] get paid more or two, we go somewhere where we get paid the same with less work to do.”

#### Theme 3: High and Complex Caseloads

Educators reported having caseloads with a high number of children with special needs or challenging behaviors. An educator shared, “… it’s very difficult when you have a 1 to 10 ratio especially if you have 5 children with behavioral issues in there [classroom, they] all need one-on-one care.” Teachers expressed a high need for support and specific training to handle challenging behaviors, and smaller classroom sizes that would account for the number of children with challenging behaviors. Educators stated that often children required constant surveillance, particularly if they have difficult behaviors that can be disruptive to a class lesson. Educators stated Head Start Performance Standards prohibit the suspension and expulsion of children which results in an increased number of children with challenging behaviors without additional personnel in the classrooms to assist. While mental health consultants often support teachers, many felt it was not enough help and voiced the need to have additional personnel to assist in the classroom.

#### Theme 4: Lack of Recognition from the K-12 Education System

Educators expressed a general lack of recognition from the traditional K-12 education system and reported feeling their work was not valued. Many reported a hope of receiving more recognition from the K-12 education system as well as from parents. Educators describe the critical role they play in setting the foundation for children’s learning and development, and believe the tide is slowly changing towards improved appreciation and value for their work and contributions. An educator shared, “So once the districts and once everyone starts noticing that we do matter, we’re the first ones that they come to, we’re the ones who’s teach them how to sit in class, socialize, we’re doing all of that.”

#### Theme 5: Poor Onboarding/Teacher Preparation Practices

There was variability with onboarding practices reported among the participants, with educators reporting that new teachers are not appropriately trained, while others reporting strong onboarding practices. In addition, deficits in teacher preparation programs were cited as another reason new teachers have difficulty adjusting to the work demands. Educators expressed that a lack of orientation and support for new educators leads to unnecessary frustration and a difficult adjustment period.

#### Theme 6: Limited Voice and Participation

Educators expressed frustration with constant change in the work-place and a lack of voice during the planning of change. Educators stated they were upset by the constant changes in curriculum, worksites, environmental factors, caseload, procedures, and new initiatives from year to year. An educator shared, “So you thought you were going to do this one year, but then the next year it’s something totally different, just out of the blue.” Educators felt the nature of their work had changed over time and expressed frustration with not having a voice that could impact their work, including curriculum development, classroom setup, and individualization. Educators were often told about the change, but were not involved in the planning process, were not asked to provide input, and did not have a voice in shaping the practice to be implemented.

### Survey Results

**Fig. 1:**
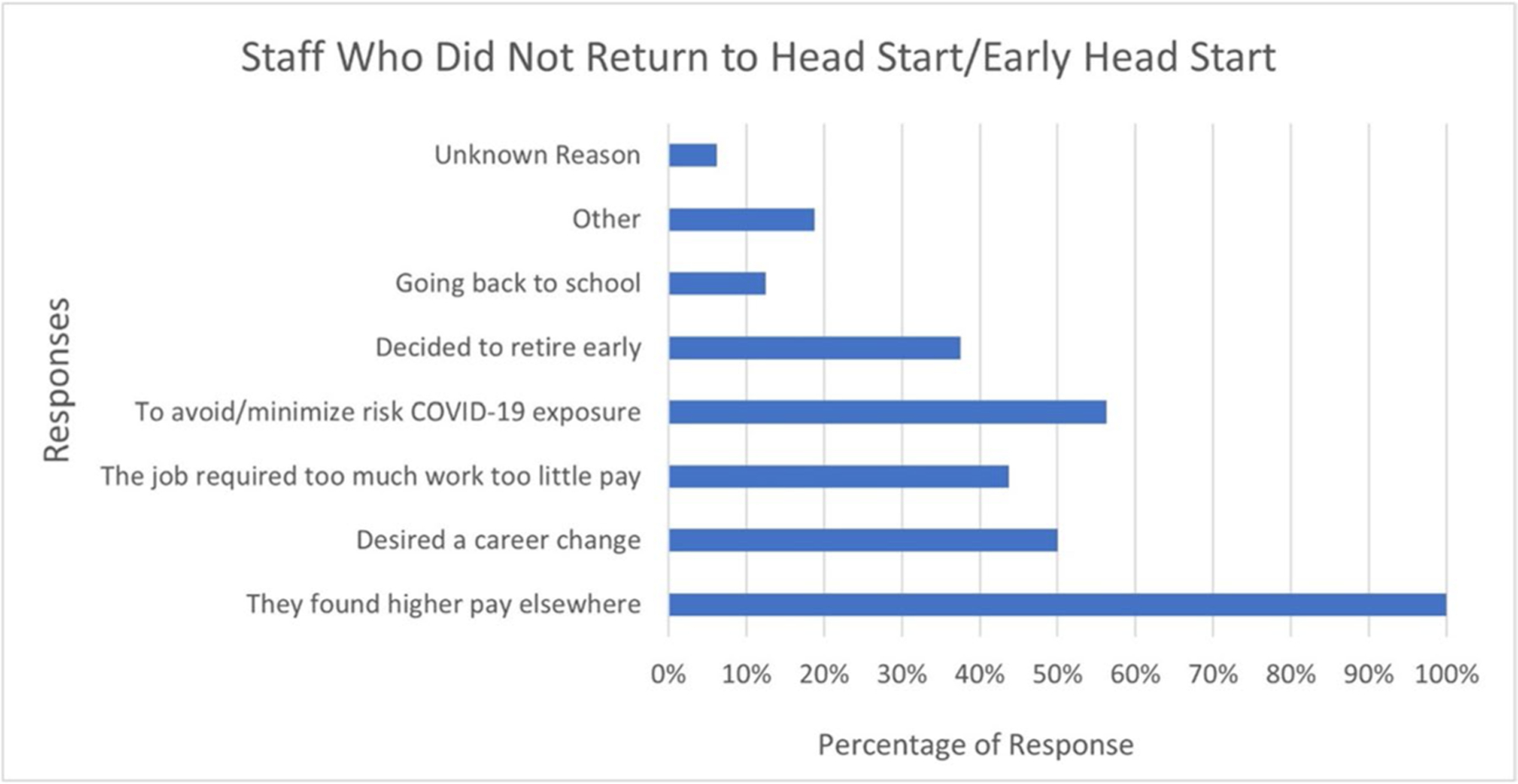
Turnover reasons provided by staff who left head start/early head start (n = 16). *Note:* This graph shows the reasons staff provided when they left a Head Start or Early Head Start program

Ninety-four percent of survey respondents reported staff shortages. Regardless of the time period (past 1, 6, or 12-months) teachers, assistant teachers, home visitors, and nutrition services staff had the highest number of vacancies while building maintenance staff and registered dietitians had the least. Higher pay elsewhere was the most common reason identified by respondents as to why staff did not return to their jobs one year after the declaration of the COVID-19 global pandemic. Avoiding and minimizing COVID-19 exposure, retiring early, or for personal reasons (moving, family responsibilities) were the most common reasons after low pay, see Fig. 1. Eighty-two percent reported having any unfilled EHS/HS spots, and 35% reported having at least 20% or more unfilled spots in the last 12 months. Participants were also asked whether their EHS/HS programs would be able to have full infant and child enrollment in the upcoming months (i.e., Jan 2022), and 24% either agreed or strongly agreed with the statement.

## Discussion

Through a secondary analysis of focus group and survey data, this study aimed to explore retention and turnover through the voices of HS and EHS education staff in light of current staff shortages. Educators reported several reasons driving their decision to stay with an HS or EHS program. Retention reasons included the satisfaction and belief that their work was making a positive difference, the impact on lives of children and families, readily available opportunities for professional development, and the collaborative relationships and strong connectedness with colleagues. Despite these reasons, however, educators also pointed out that low wages, high volumes of paperwork, lack of staff and support for children with challenging behaviors in the classroom, a lack of recognition from the K-12 educational system, weak onboarding or teacher preparation practices, and not having input or participation in EHS and HS programs were all reasons they considered to be staff turnover drivers. These findings add richness to the limited literature on reasons for retention among EHS and HS staff, and factors for turnover not previously described. Collectively, these can be used to leverage the retention of EHS and HS educators to prevent further staff shortages.

According to Alderfer’s (1969) ERG theory, employees need to fulfill three human core needs *existence*, *relatedness*, and *growth*. The voices of EHS and HS staff reaffirmed ERG theory and indicate that if their *growth* and *relatedness* needs were being met, they were more likely to stay in the organization despite the low wages. EHS and HS educators stated they were unlikely to leave if they perceived their work to be making a meaningful impact or difference in the lives of children and families. Consistent with previous research, preschool teachers’ intrinsic motivation for teaching is due to their commitment to working with children and contributing to the social good (Bullough et al., 2012; McDonald et al., 2018; Wells, 2015). In addition, staff voiced an intent to stay in the organization when their relatedness needs were met through a positive workplace climate, confirming previous findings (Grant et al., 2019; Wells, 2015). However, this study identified that a positive workplace was defined not only by policy and leadership, but also perceived strong positive work-relationships with co-workers. In contrast, educators reported intentions to leave the workplace when they lacked positive relationships with others in the workplace. Our findings are consistent with previous literature that found, that in addition to improved pay and salary, retention factors among early education staff include health insurance benefits, feeling supported in the workplace, and working conditions perceived as equitable, fair, and positive (Bryant et al., 2023; Capone & Petrillo, 2016; Jeon & Wells, 2018; Schaack et al., 2022) Thus, fulfilling *growth* and *relatedness* human needs through organizational practices can outweigh the drivers of turnover.

Educators, however, also pointed out that poor wages are a strong driver of leaving Head Start despite having the “heart and passion” for the job and enjoying their workplace environment and colleagues. Early childhood educators’ *existence* needs are not being met through Head Start compensation. Head Start staff voiced that they are unable to meet their meet basic needs without the support of a partner or a second or third job. For many staff this translated to working more than 60 hours a week. Our quantitative data also showed that higher pay elsewhere was the most common reason as to why EHS and HS staff did not return to work during the COVID-19 pandemic and contributed to the staff shortages. These findings are consistent with previous research, which shows low salary as a major contributor to turnover among early childhood educators and many falling short of a livable wage in most states (Hale-Jinks et al., 2006; Whitebook et al., 2014; Whitebook & Sakai, 2003; McLean et al., 2021).

Despite the research and decades of knowledge that the early childhood workforce is among the lowest paid, low wages are still problematic among workers in early childhood educational settings (Hale-Jinks et al., 2006; McLean et al., 2021; Phillips et al., 2016; Whitebook et al., 2014; Whitebook & Sakai, 2003). Not addressing unlivable wages is also problematic when these caring professions are not economically recognized for their work and are paid low relative to the education and skill requirements (England & Folbre, 1999). While early childhood education is recognized to be an important determinant of healthy growth and development among children, and a strong predictor of future learning, the field has neglected to equitably compensate those who provide the service. While the educational requirements have increased over time, including requiring Head Start teachers to have bachelor’s degrees, the economic reward has been minimal. There is a need and urgency to develop policies and practices that offer an equitable salary based on education, experience level, and local cost of living; otherwise, there is a substantial risk Head Start programs may not recover from an already high number of unfilled job vacancies and continue to lose great teachers with exceptional training to school districts or other higher-paying organizations.

Through the voices of Head Start staff, we learned training provided by Head Start programs is exceptional and many reported being prepared for future career growth. Thus, the Head Start workforce is highly qualified (Office of Head Start, 2023). Staff who stayed in Head Start often left the classroom to move into administrative positions in order to increase their salary. Thus, salary increases for EHS and HS educational staff can be a strong driver of retaining qualified teaching staff (Bridges et al., 2011), and low salaries may lead to great teachers leaving the classroom. In light of Universal Pre-Kindergarten and Transitional Kindergarten policy expansions nationwide and the high qualifications of Head Start and Early Head Start staff, turnover may continue to increase if nothing is done to match skills with wage premiums.

Other turnover factors were identified by educational staff as organizational. Staff voiced dissatisfaction with the excessive amount of paperwork and completion of forms that were often outdated and perceived as irrelevant. Educators reported that paperwork was an activity that was getting in the way of teaching. Previous literature has reported that educators feel overburdened with paperwork and feared the amount of paperwork affected their quality of instruction (Bullough, 2015; Bullough et al., 2014; Harden et al., 2010; Wells, 2017). Educators also shared they were constantly adapting to new regulations, guidelines, deadlines, and teaching methodologies and as a result, confronted with constant changing practices and the added responsibility to document processes and results of program changes. Many referenced that these changes occurred yearly and expressed unawareness of why there were so many changes (Bullough et al., 2014). The degree of paperwork was often in conflict with their passion for teaching. Yet they continued to meet the increase in paperwork demands often completing it in their own time at home with their managers unaware.

This experience for educators may be a result of Head Start programs being complex open systems, which are interdependent with the federal budget, state-specific early childhood learning policies, research, and local community and educational needs (Ballet & Kelchtermans, 2008). New Head Start leadership further complicates shifting priorities requiring programs to respond and align to new goals. Consistent with system’s theory, program administration often implements changes that respond to macrolevel shifts which may contribute to fluctuations in workload, paperwork, and overall duties to an already existing heavy workload. The outcome of the call for change or improvement often results in work intensification for teachers, and teachers intrinsically committed to child outcomes and their jobs implement the change (Ballet & Kelchtermans, 2008) without assistance, additional compensation, often investing long hours. An additional effect of macrolevel shifts and the impact on interdependent parts of Early Head Start and Head Start programs is staff often feeling voiceless or powerless to object to changes in responsibilities (Bullough et al., 2014). Head Start administrators, therefore, must balance macrolevel shifts that impact internal systems and identify practices and ways to increase transparency and collaboration to adapt to the changes. Doing so may provide a buffer from the overwhelming and unsatisfying experiences many staff face and should be a high priority for HS organization who will continue to experience changes in the early childhood education landscape.

### Recommendations and Suggestions

In efforts to promote retention and prevent turnover, the following are program-level recommendations.

- Emphasize the positive impact Head Start staff has on children and family outcomes.
- Include education staff in the interview process of potential hires to promote a strong culture of comradery, connectedness, and inclusivity.
- Create and foster teambuilding opportunities.
- Pursue equal pay that is comparable to kindergarten teachers and accounts for the cost of living in their respective states and counties.
- Review data tracking systems to identify irrelevant data collection and reduce the paperwork burden.
- Ensure lines of communication are strong between administration and staff, with opportunities for staff input in programming changes.
- Evaluate the workload when new initiatives are added with consideration of the number of changes and develop transition plans.

### Limitations

There are limitations to this study. This study describes the current views of Head Start staff on turnover and retention, and these may change over time. The sample included Head Start and Early Head Start programs servicing a few states, which may have hindered specific-county or community findings. We did not collect the perspective of educators who have left Head Start or Early Head Start. In addition, the survey and focus groups sampled a small group of Head Start leaders and staff, which may not be generalizable to other programs.

### Conclusion

In the field of early childhood education, teacher turnover has a significant impact on children, families, and supporting staff. In order to retain high-quality early childhood educators in Head Start programs it is imperative to provide competitive wages, consider reducing workload and paperwork, value the positive impact Head Start staff has on children and families, and include educators in planning phases to programmatic changes. Establishing workforce practices that prioritize and emphasize these areas may facilitate retention. With the ongoing shortages of teaching staff, responding to the drivers of turnover, and building upon the strengths and retention factors of the Head Start workforce are urgently needed in order to provide all children with equitable learning and development outcomes.

## Figures and Tables

**Table 1:** Demographics and characteristics of head start education staff (N = 23)

Characteristics of Focus Group	N (%)
Gender: Female	21 (91%)
Race/Ethnicity	
Hispanic/Latinx	8 (35%)
White	7 (31%)
Black/African American	6 (26%)
Asian	1 (4%)
American Indian	1 (4%)
Highest Level of Education	
Some college	2 (9%)
Associate degree	5 (22%)
Bachelor’s degree	12 (52%)
Graduate degree	4 (17%)
Years Employed at Current Program, mean	8.7
Less than 5 years	11
5–10 years	2
10–20 years	6
More than 20 years	4
Years in Early Childhood Education Field, mean	18.9
Less than 10 years	5
10–20 years	7
More than 20 years	11
Agency Type	
Non-Profit	12 (52%)
Local Education Agency	10 (43%)
Tribal Program	1 (4%)

This table describes the demographics of the focus group participants

## Data Availability

Data sharing is not applicable to this article as the authors received the data for secondary analysis.
